# Long-term use of cover crops and no-till shift soil microbial community life strategies in agricultural soil

**DOI:** 10.1371/journal.pone.0192953

**Published:** 2018-02-15

**Authors:** Radomir Schmidt, Kelly Gravuer, Anne V. Bossange, Jeffrey Mitchell, Kate Scow

**Affiliations:** 1 Department of Land, Air and Water Resources, University of California, Davis, Davis, California, United States of America; 2 Department of Plant Sciences, University of California, Davis, Davis, California, United States of America; Leibniz-Institute of Vegetable and Ornamental Crops, GERMANY

## Abstract

Reducing tillage and growing cover crops, widely recommended practices for boosting soil health, have major impacts on soil communities. Surprisingly little is known about their impacts on soil microbial functional diversity, and especially so in irrigated Mediterranean ecosystems. In long-term experimental plots at the West Side Research and Extension Center in California’s Central Valley, we characterized soil microbial communities in the presence or absence of physical disturbance due to tillage, in the presence or absence of cover crops, and at three depths: 0–5, 5–15 and 15–30 cm. This characterization included qPCR for bacterial and archaeal abundances, DNA sequencing of the 16S rRNA gene, and phylogenetic estimation of two ecologically important microbial traits (rRNA gene copy number and genome size). Total (bacterial + archaeal) diversity was higher in no-till than standard till; diversity increased with depth in no-till but decreased with depth in standard till. Total bacterial numbers were higher in cover cropped plots at all depths, while no-till treatments showed higher numbers in 0–5 cm but lower numbers at lower depths compared to standard tillage. Trait estimates suggested that different farming practices and depths favored distinctly different microbial life strategies. Tillage in the absence of cover crops shifted microbial communities towards fast growing *competitors*, while no-till shifted them toward slow growing *stress tolerators*. Across all treatment combinations, increasing depth resulted in a shift towards *stress tolerators*. Cover crops shifted the communities towards *ruderals*–organisms with wider metabolic capacities and moderate rates of growth. Overall, our results are consistent with decreasing nutrient availability with soil depth and under no-till treatments, bursts of nutrient availability and niche homogenization under standard tillage, and increases in C supply and variety provided by cover crops. Understanding how agricultural practices shift microbial abundance, diversity and life strategies, such as presented here, can assist with designing farming systems that can support high yields, while enhancing C sequestration and increasing resilience to climate change.

## Introduction

Reducing tillage and growing cover crops, widely recommended practices for boosting soil health, are known to impact soil microbial communities; however, we know far less about impacts on biology than on physical and chemical properties of soil. [1,2,3]. Reduction or elimination of tillage can improve soil structure and water holding capacity [4,5], and cover crops increase C and N inputs through plant residue decomposition, root exudates and symbiotic N fixation [1,6].

Benefits and costs of cover cropping and reduced tillage are dependent on the particular agronomic context. For example, cover crops can improve crop yield stability, primarily by increasing soil water infiltration and storage capacity [4,5]. They can reduce erosion, increase microbial biomass and soil C and N, control weeds and help prevent excess nutrients leaching into groundwater [7,8,9,10]. However, cover crops can also incur higher financial and managements costs and in drier climates may reduce water available to subsequent crops [1,11]. Also, decomposition of cover crops must be rapid enough to allow timely planting of the following crop [1,12]. No-till farming can reduce costs by reducing the use of heavy and expensive equipment, and can increase soil aggregation, SOM in the surface layer, water infiltration, water holding capacity, and reduce erosion [11,12,13,14]. On the other hand, no-till fields require closer monitoring, increased herbicide use for weed management, specialized equipment for planting new crops in surface residue, and may lead to reduced yields [1,15,16,17,18].

Microorganisms are critically important to maintaining the physical structure and many functions of soil, yet surprisingly little is known about how cover cropping, and tillage impact soil microbial community composition and the services they provide in agroecosystems [19]. Measures of overall community composition, such as DNA sequencing of taxonomic marker genes, are frequently used in studies aiming to link microbial community structure to function, yet these approaches have so far been of limited practical value (see e.g. [9,20,21]) due to gaps in our understanding of how phylogenic patterns relate to important soil functions [22,23]. To assess whether changes in community composition represent shifts among ecologically distinct types of microbes or merely substitutions of taxa that are ecologically similar, putting greater focus on measuring ecologically important microbial traits holds promise [24]. Understanding how management changes the soil environment and its interactions with microbial communities can provide a foundation for rational design of improved agricultural systems.

In bacteria, two traits hypothesized to have ecological importance are 16S rRNA gene copy number and genome size. Ribosomal gene copy numbers correlate with the speed of resource use—fast growing microbes that quickly utilize nutrient pulses tend to have more copies of ribosomal genes than slow growing microbes adapted to a steady supply of low level nutrients [25,26,27]. In contrast, genome sizes relate to the range of resource use—specialist organisms relying on a narrow range of C sources, whether fast growing or slow growing, often have smaller genomes than generalists that can utilize a wide range of resources [28,29].

The Competitor-Stress tolerator-Ruderal life strategy scheme (C-S-R) designed for plant communities [30], provides a framework to help link ecologically important traits to overall community function. The C-S-R framework places organisms in a continuum that encompasses three distinct life strategies: a) rapid resource utilization in productive environments (competitors), b) persistence under unfavorable, resource-limiting conditions (stress tolerators), or c) re-colonization and persistence under conditions of frequent disturbance (ruderals) [30,31,32]. This approach has been successfully applied in classifying methane-oxidizing bacteria in soil according to their phylogenetic and functional properties [33]. In addition, because the C-S-R approach allows classification of microbes employing mixed life strategies (e.g., CS, SR, etc.), it offers flexibility to accommodate the vast metabolic diversity of bacteria [31].

The effects of tillage and cover cropping have been studied extensively in temperate, rain fed systems, but their impacts on soils in irrigated Mediterranean systems have received far less attention [11] and might be expected to differ due to the absence of a freeze-thaw cycle and fluctuations in water availability throughout the year. A field scale no-till and cover cropping experiment has been in place at the UC Davis West Side Research and Extension Center (Research Center) at Five Points, CA since 1999 [11]. Reduced tillage and/or cover cropping treatments increased total soil C by 12–53% and total soil N by 10–47% in the top 30 cm of soil compared to standard tillage systems with no cover crops [12]. Reduced tillage or cover cropping was also associated with 66–147% higher water-stable aggregates, better water infiltration and reduced slaking compared to standard tillage systems in the top 15 cm of the soil [34]. Combining reduced tillage with cover cropping had the greatest impact on soil health parameters [34,35].

We hypothesized that:

1. Microbial communities in different systems will vary in their operational taxonomic units (OTUs) and life strategy composition due to farming management practices.
2. No-till and cover crop plots will have higher microbial abundance and diversity due to more carbon and greater heterogeneity in microenvironments;
3. The greater supply of nutrients in cover crop treatments will favor competitors;
4. Stress tolerators will be favored in no-till treatments and at deeper depths in all systems due to lower nutrient availability; and
5. Tilled plots will have greater relative abundance of ruderals given the disturbance due to tillage and associated pulsed nutrient availability.

Using qPCR, DNA sequencing of the 16S rRNA gene, and phylogenetic estimation of ecologically important microbial traits, we compared cropland soil microbial communities in the presence or absence of tillage, with or without cover crops, and at three depths: 0–5, 5–15 and 15–30 cm.

## Materials and methods

### Site description

The 427 m by 100 m study site is located at the University of California’s West Side Research and Extension Center (WSREC - http://ucanr.edu/sites/westsiderec) in Five Points, CA (36°20′29″N, 120°7′14″W). The soils are Panoche clay loam (fine-loamy, mixed superlative, thermic Typic Haplocambids) (Arroues, 2006). Before the onset of experimental treatments (1998), a uniform barley (*Hordeum vulgare* L.) crop was grown and removed as green chop silage to reduce differences in soil water and fertility that may have existed due to previous research. Four experimental treatments were implemented for 15 years prior to soil sampling for our study: no-till no cover crops (NTNO), no-till plus cover crop (NTCC), standard tillage no cover crops (STNO), and standard tillage plus cover crop (STCC) in a drip irrigated tomato/cotton rotation. Both rotation crops were grown simultaneously, one in each half of the experimental field. Each treatment was applied in four replicate plots in a semi-randomized block design, for a total of 32 plots in 8 blocks. Each block contained all four experimental treatments. This study was conducted on the southern half of the no-till research plots, specifically plots 1–16 that were under tomato crop in 2013 (S1 Table).

### Soil sampling and characterization

Soil physicochemical data have been collected regularly from 1999 to 2013 [11,12]; fall 2013 data were used in the analyses below. Soils were sampled at two depths (0–15 cm and 15–30 cm) in the fall after harvest [11,34,35]. Briefly, in each plot, six to eight 7.6-cm-diameter cores per depth were composited before air drying, sieving through a 2 mm sieve and grinding using a soil pulverizer to pass through a 60 mesh screen, and dried to constant weight according to protocols of the University of California, Davis, Analytical Laboratory (http://anlab.ucdavis.edu/sampling/soil-sampling-and-preparation). Total C and total N were measured using a combustion C analyzer (CE Elantech, Inc., Lakewood, NJ). Bulk density was measured by the compliant cavity method [36]. Surface soil water stable aggregate percentages, and water infiltration were determined using USDA NRCS Soil Quality Test Kit procedures [37].

For microbial characterization, soil samples were collected from plots 1–16 on 22/11/2013. Six 2.5 cm diameter cores were collected from each of three depths (0–5, 5–15 and 15–30 cm) at each plot. The six samples from each plot/depth were homogenized and placed on ice in the field, then stored at -20°C before further analysis.

### Tillage and cover cropping treatments

The ST and the NT systems are described in detail in [11,38]. Briefly, standard tillage (ST) consisted of residue shredding, multiple diskings to incorporate residues and break up soil clods, listing of beds, and power incorporation of the surface 10 cm of soil using a cultimulcher (BW Implement, Buttonwillow, CA). The no-till (NT) systems management included controlled traffic farming, and planting beds were not moved or destroyed during the entire study period. Following tillage operations, surface residue typically averaged over 90% for the NTCC, between 40 and 70% for the NTNO, between 10 and 20% for the STCC, and below 5% for the STNO [11].

The tomato and cotton crops were furrow irrigated from 2000–2012. In keeping with trends in the region, the study site was converted to subsurface drip irrigation in 2013 with 2.2 cm diameter tape buried 30 cm deep in the centers of each 1.5 m-wide planting bed with 30 cm emitter spacing. Installation of the drip tape constituted a tillage operation to all systems.

A CC mix of Juan triticale (*Triticosecale* Wittm.), Merced rye (*Secale cereale* L.) and common vetch (*Vicia sativa* L.) was seeded using either a 4.6 m John Deere 1530 no-tillage single-disc opener seeder (Moline, IL) or a 4.6 m Sunflower 1510 double-disc opener no-till drill (Beloit, KS) at 19 cm row spacing and at a rate of 100 lbs ac^-1^ (30% triticale, 30% rye and 40% vetch by weight) in late October in the STCC and NTCC plots and irrigated once with 10 cm of water in 1999 and again with 5 cm in 2012. The legume species was inoculated with rhizobium before seeding. From 2000 to 2011 and in 2013, no irrigation was applied to the cover crops, which were planted in advance of winter rains. Between 2010 and 2013, the basic CC mixture was changed to include a greater diversity of species including pea (*Pisum sativum* L.), faba bean (*Vicia faba* l.), radish (*Raphanus sativus*), and Phacelia (*Phacelia tanacetifoli*) [11].

### DNA extraction

Soil DNA was extracted in triplicate from 0.25 g (total humid weight) of soil using the Power Soil DNA Isolation Kit (MoBIO Laboratories, Carlsbad, CA, USA), according to the manufacturer’s instructions. The quality and relative quantity of the extracted DNA was determined using a Qubit colorimetric assay apparatus (Invitrogen, NJ, USA).

### qPCR

The qPCR was performed on an Applied Biosystems (Applied Biosystems, NJ, USA) ABI 7300 sequence detection system using SYBR Green detection. The qPCR was performed in 20 μL reaction mixtures containing 10 μL of SYBR GreenER^™^ qPCR SuperMix (Invitrogen, NJ, USA) and 0.5 μM of each primer. Gene amplification was carried out with primer set 341F/534R for bacterial 16S rRNA gene [39,40,41] and with primers Arch771F/957R for Archaeal 16S rRNA gene [42].

A melting curve analysis was performed after each assay to ensure that only the products of the desired melting temperature were generated from the SYBR green qPCR. The *R*^*2*^ values for the standard curves were 0.99 or better for all runs. All reactions were run in triplicate with a standard curve spanning 10^1^–10^6^ copy numbers for bacterial and archaeal 16S rRNA genes. The standard curves for quantifying gene copy numbers were determined by cloning the PCR products in a plasmid using the procedures reported by Okano et al. (2004). The population sizes of total bacteria and total archaea were estimated as the normalized copies per gram of dry soil.

### Sequencing

Amplification of the V4 hypervariable region of 16S rDNA was carried out using primer pair F515 (59-CACGGTCGKCGGCGCCATT-39) and R806 (59-GGACTACHVGGGTWTCTAAT-39) as described by [43] designed to include Illumina adaptor and barcode sequences (S2 Table). Amplicons were mixed at roughly equivalent ratios based on electrophoretic band intensity and purified using Agencourt AMPure XP PCR purification system (Beckman-Coulter, CA, USA). Pooled samples were submitted to the University of California Davis Genome Center for 250-bp paired-end sequencing on the MiSeq platform. The total sequence count was 5379092 sequences. Raw Illumina fastq files were demultiplexed, quality filtered (Q30), and analyzed using QIIME 1.9 and the GreenGenes 13.5 reference database. QIIME was used to assign Operational Taxonomic Units (OTUs) using UCLUST, with a threshold of 97% pairwise identity. OUT tables were rarefied to 4000 sequences per sample for further analysis. Of these, 99% were identified to phylum level, 0.02% were identified as bacterial sequences but not assigned at phylum level and 0.98% were classified as non bacterial/archaeal. OTU richness as calculated in QIIME was used to estimate Alpha diversity. Unweighted Unifrac distances were used to estimate Beta diversity. Raw sequence data was submitted to the NCBI Sequence Read Archive (SRA) (https://www.ncbi.nlm.nih.gov/sra)–project accession number PRJNA353955.

### Trait value estimation

Trait estimation and statistical analyses were conducted using R statistical software [44] in RStudio version 0.99.446 (RStudio, Inc. 2015). Trait values for each OTU were estimated using a reference tree of bacteria and archaea with known trait values, and the reliability of the trait estimates was tested as described in Gravuer and Eskelinen (2017) [45]. Briefly, OTUs were placed onto the reference tree using pplacer software [46] and rRNA gene copy number and genome size values were estimated for each placed OTU using picante:phyEstimate [47]. This method does not directly rely on OTU taxonomic assignments to estimate trait values; rather, pplacer selects the best-supported phylogenetic placement of each OTU on the tree of reference genomes, and phyEstimate uses ancestral state estimation techniques to estimate the OTU’s trait value based on the known trait values of multiple surrounding reference taxa. Using the copy number estimates, relative abundances in the initial OTU table were adjusted with the script from Kembel et al. (2012) [48]. Community weighted mean trait values were then calculated for each trait in each sample with the FD package [49,50] using this adjusted OTU table. Microbial communities were assigned to the C-S-R life strategy framework conceptual model based on direct comparisons of estimated trait values.

### Statistical analysis

Linear mixed models (R package nlme [51]) were used to model community-weighted mean trait values, as well as abundance of bacteria and archaea, as a function of depth, tillage, cover cropping, and all interactions as fixed effects with plot as a random effect. Canonical Correspondence Analysis (CCA) (R package Vegan [52]) was used to model community composition constrained by soil physicochemical parameters. Data for total bacteria and archaea were log transformed for analysis to meet the assumption of homogeneity of variance.

## Results

### Soil chemical properties

There were three patterns of changes in soil physicochemical properties in the top 30 cm of soil, as described previously [11]; briefly: 1) pH, EC, P, NO_3_^-^ exhibited some seasonal changes but no trends over time or significant differences between treatments were observed; 2) organic matter (OM) increased in all plots, but significantly higher increases were observed in cover crop (CC) than no cover crop (NO) treatments irrespective of tillage; 3) in the no cover crop treatments, total C and N showed no change while K showed a slight decrease, whereas in the CC treatments all three parameters increased in value. Differences between treatments were greater at 0–15 cm than at 15–30 cm depths. Total bacteria numbers were positively correlated with OM, P, K and total N and C; total archaea numbers were positively correlated with total bacteria numbers, P, K and total N and C (S3 Table). In addition, total N and C were positively correlated with P and K (p < 0.05).

### Microbial abundance

**Fig 1:**
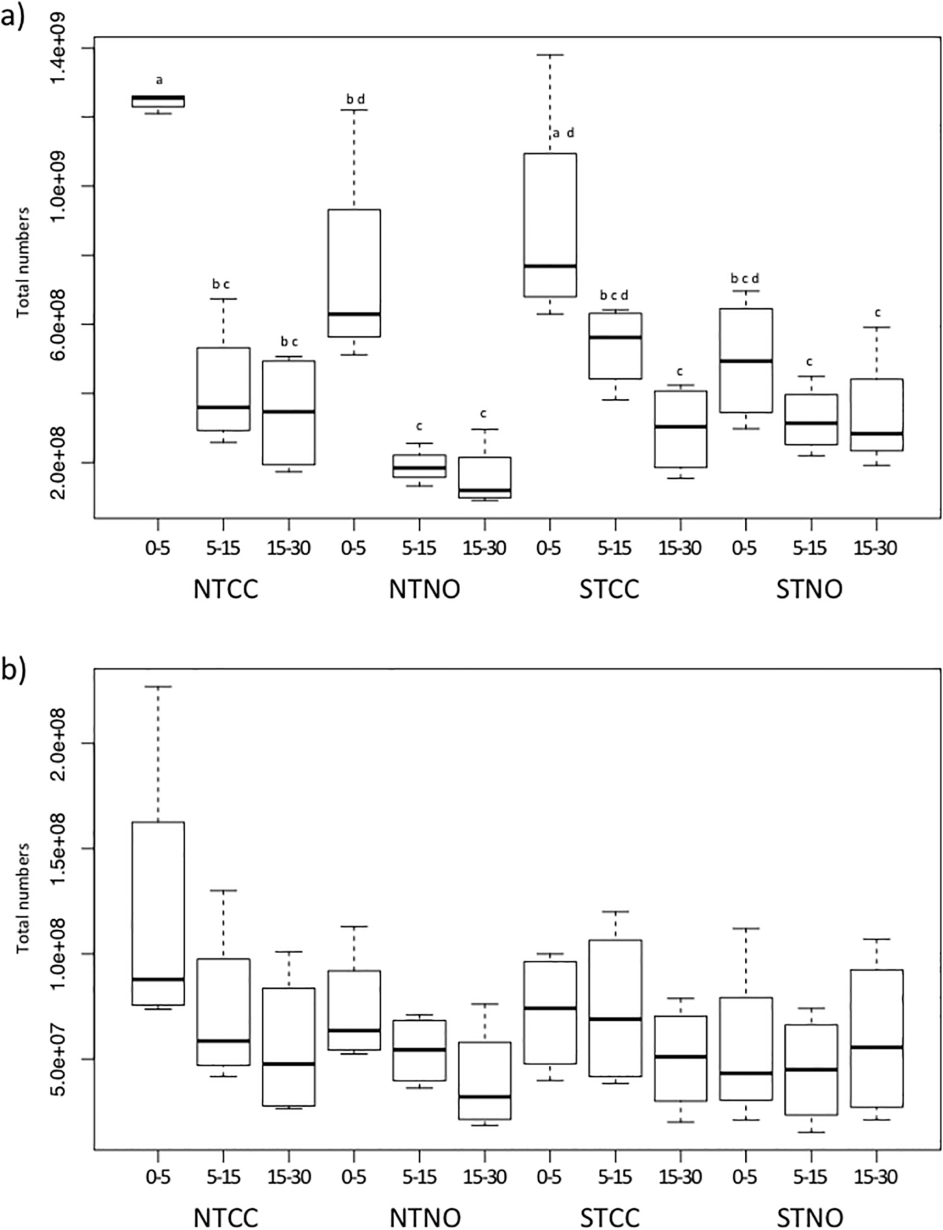
Total microbial numbers in a Mediterranean-climate agricultural soil at a combination of different depths and under different cropping regimes. a) total bacteria and b) total archaea. Depths – 0–5, 5–15, 15–30 cm. NTCC—cover crop, no-till; NTNO—no-till, no cover crop; STCC—standard till, cover crop; STNO—standard till, no cover crop. Letters above boxplots indicate significant difference (p < 0.05); no significant difference was observed for archaea numbers.

Microbial abundance changes with depth depended on treatment, with large decreases in bacterial numbers under no-till between the 0–5 to 5–15 cm depth, yet little change between the 5–15 and 15–30 cm depth. Under standard tillage in the absence of cover crops bacterial numbers were similar at all three depths (Fig 1). Interestingly, the standard till cover cropped treatment showed a gradual decrease in bacterial numbers through the soil profile somewhat similar to the no till treatments, but without the rapid decrease in numbers below the top 5 cm layer. Cover cropping led to a significant higher total bacterial numbers in comparison to NO treatments (p = 0.008) (S2 Fig).

Archaea showed similar trends to bacteria overall (Fig 1) with increased numbers with cover crops (p = 0.02) (S2 Fig). The magnitude of change in numbers between NO and cover crop treatments was lower for the archaea than for bacteria. No significant difference was observed with tillage alone for either total bacteria or archaea (S2 Fig). Overall, bacterial numbers were more sensitive to differences in treatments than were archaea (Fig 1). Archaea numbers increased as relative proportion of total microbes with increasing depth in all treatments. Archaea also showed a strong block effect, not evident with bacteria (S4 Table).

### Microbial composition and diversity

**Fig 2:**
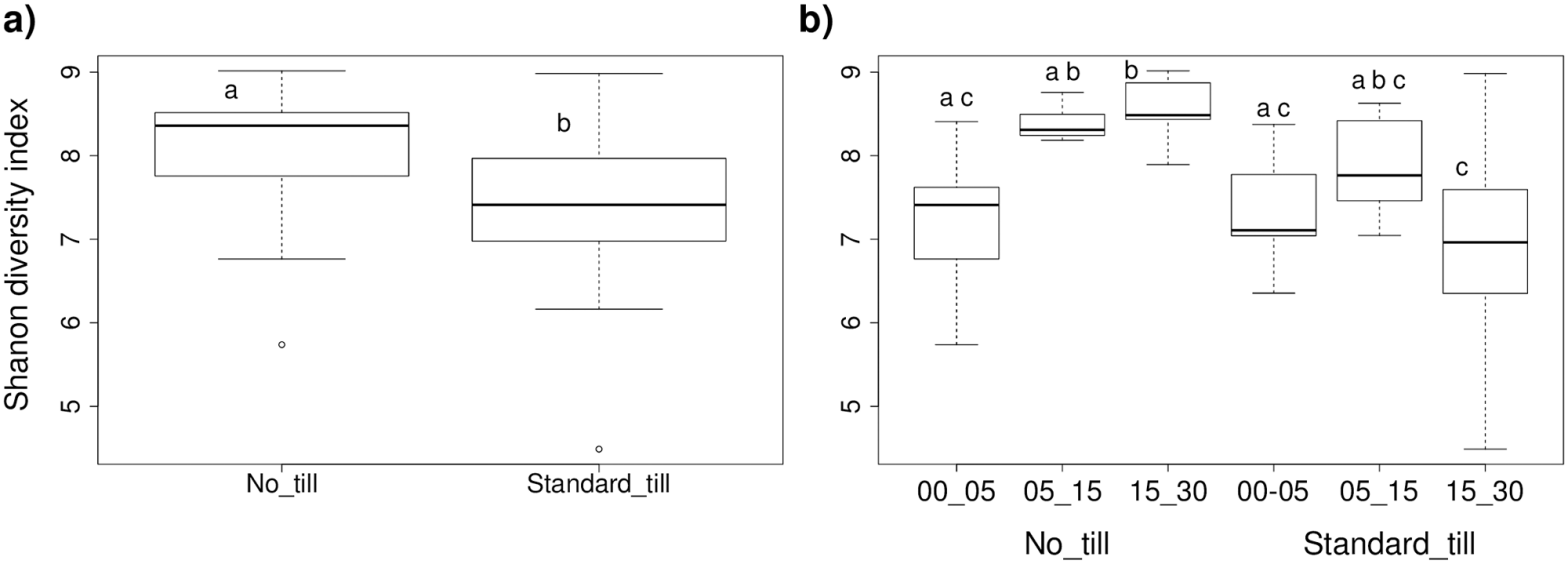
Variation in microbial community diversity under different management systems. a) different tillage treatments; b) different depths and tillage treatments. (Shannon diversity index). Letters above boxplots indicate significant difference (p < 0.05).

Microbial diversity, measured by Shannon Index, was significantly different in till versus no-till, and till versus no-till in combination with depth (Fig 2). Overall diversity was greater under no-till, and increased with increasing depth. Under standard till, diversity decreased with depth (Fig 2b).

**Fig 3:**
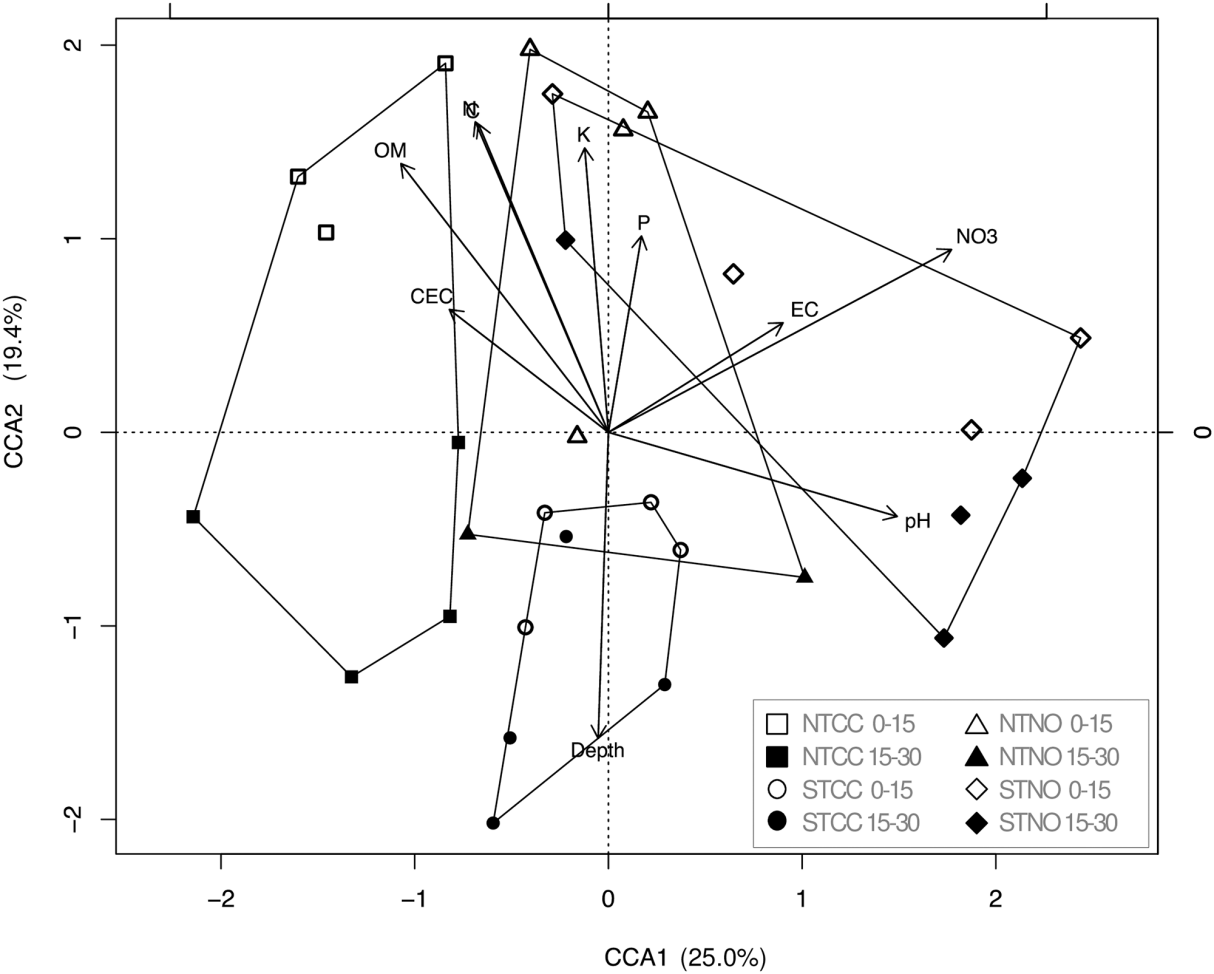
CCA analysis of microbial sequence data. Canonical correlation analysis (CCA) of 16S rRNA gene sequence data identified to genus level constrained by soil physicochemical characteristics.

Soil physicochemical properties and phylogenetic composition both contributed to the differentiation of bacterial communities under the four treatments, with the STNO community most distinct from the other three treatments along the first axis (Fig 3). The two cover cropped treatments converged with the STNO treatment at 0–15 cm depth, but all three treatments separated further from each other at 15–30 cm depth. These treatments were also associated with increased amounts of OM, C, N, P and K at the shallow depth. The NTNO treatment separated from all other treatments along the second axis and was most strongly associated with soil depth.

Overall, the dominant phyla (with *Proteobacteria* considered in their individual classes) across all systems were *Actinobacteria* (27.2±6.4%), *Acidobacteria* (12.8±4.7%), *Betaproteobacteria* (10.3±6.1%), *Chloroflexi* (8.7±2.4%), *Alphaproteobacteria* (8.0±2.0%) and *Planctomycetes* (7.6±2.2%) (S5 Table). Of the top 15 bacterial phyla, the only group that showed a significant response to tillage was the *Firmicutes*, with their relative proportion increasing under standard till (ST) treatments (S5 Table). *Alphaproteobacteria*, *Verrucomicrobia* and *Deltaproteobacteria* fractions increased with cover crops, while *Firmicute* fractions increased in the absence of cover crops (S5 Table). *Bacteroidetes* and *Verrucomicrobia* fractions decreased with depth, whereas, in contrast, *Chloroflexi*, *Nitrospirae* and *WS3* fractions increased at 15–30 cm depth (S5 Table).

### Trait analysis

**Fig 4:**
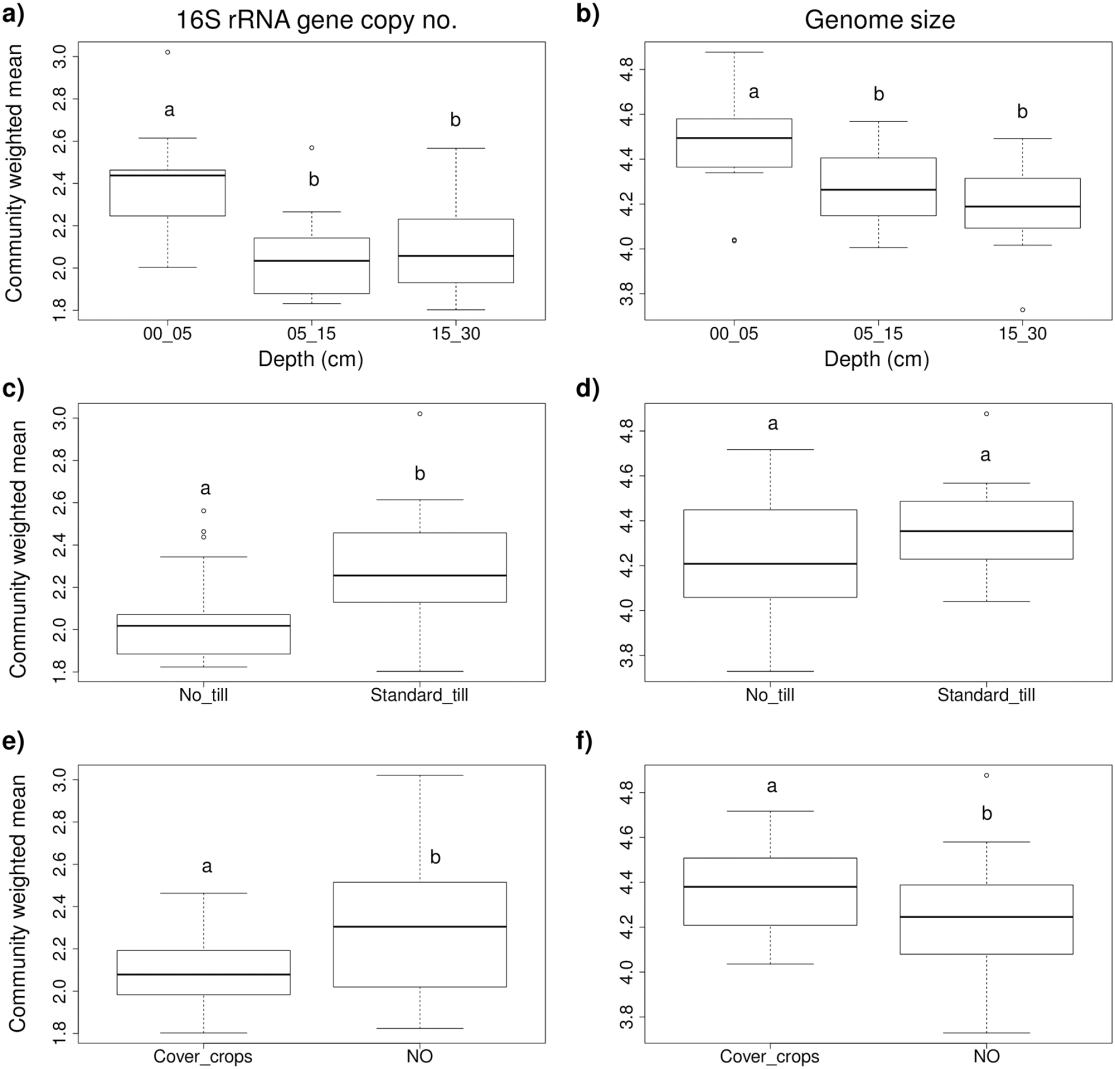
Estimation of ecologically important traits in Mediterranean-climate agricultural soils under different management regimes. The effect of different treatments on community-weighted mean estimated traits (rRNA gene copy numbers and genome size) pooled across treatments. The effects of a), b) depth; c), d) tillage; e), f) cover cropping are shown. Letters above boxplots indicate significant difference (p < 0.05).

**Fig 5:**
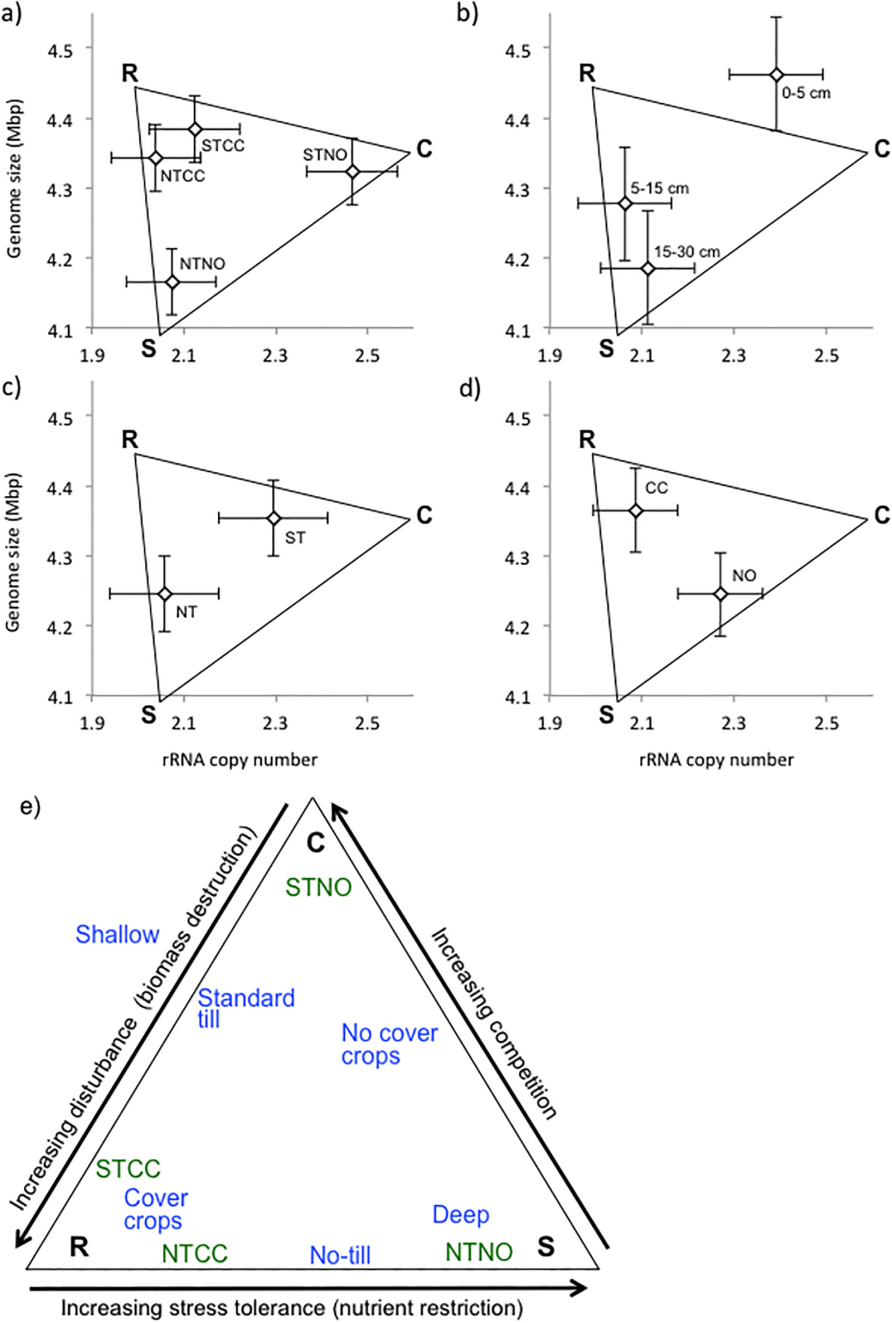
Community-weighed mean rRNA gene copy number vs mean genome size. Distribution according to average values for a) treatment; b) depth; c) tillage; d) cover crops. Error bars represent 1 standard error; e) Distribution of treatments within the Competitor-Stress tolerator-Ruderal (C-S-R) life strategy framework based on candidate trait estimates (a-d). Framework model modified from [31].

Significant (p<0.05) differences in community-weighted mean genome size and 16S rRNA copy number by depth and treatment are shown in Fig 4 and S1 Fig. Deeper soils selected for slow growing specialists (small genomes and low rRNA copy number), tillage selected for faster growing specialists (high rRNA copy number), and cover crops selected for slower growing generalists (large genomes, low rRNA copy number). By plotting the averaged values for the community-weighted mean genome size versus 16S rRNA copy number for each of depth, cover crops, tillage, and cover crop + tillage treatment, we were able to map the treatments and depths onto the C-S-R life strategy framework conceptual model (Fig 5). The trait analysis showed broadly similar trends to the CCA. In both cases the two cover crop treatments were more similar to each other while the no cover crop treatments separated further from each other, depth was an important factor, and the NTNO treatment aligned with deeper soil characteristics (Figs 3 and 5).

## Discussion

Differences in management by no-till or regular tillage, the use or absence of cover crops, and position within the soil profile strongly influenced soil microbial communities. Cover cropped communities were similar despite differences in tillage, while tillage disturbance played a large role in diverging communities in the absence of cover crops. Community composition and function varied substantially between the surface and subsurface layers in the cover cropped and/or no-till communities, in contrast to limited differences with depth under standard till, no cover crop management.

### Effects of depth

Previous studies have shown that no-till farming practices lead to higher microbial biomass in the top soil layer than under standard tillage [53,54,55]. We found similar results for the distribution of bacteria with depth (Fig 1). The correlation between bacterial numbers and OM is consistent with carbon as the primary driver of bacterial proliferation in the soil [56,57], with most of the carbon tied up in the top soil layer in the absence of tillage [12]. Cover crops have been shown to offset the loss in microbial diversity and biomass often observed in systems using intensive mineral fertilizer application [58,59]. The results of this study, and particularly the analysis of the STCC treatment, showed that cover crops can reverse some of the effects of tillage as well, in particular in re-establishing a biomass gradient with depth (Fig 1 and S1 Fig).

Due to reliance on different life strategies, archaea can respond differently to environmental selection than bacteria [60]. The absence of a significant decrease in archaeal density with depth under cover crop and no-till treatments, in comparison to the steep decrease in bacterial numbers under the same treatments (Fig 1), may reflect lower archaeal competitiveness with bacteria at the shallow depth on one hand, and better relative adaptability to nutrient limitation at greater depth on the other [61]. Archaea are thought to be generally slower growing than bacteria, and therefore only successfully compete with bacteria in niches where chronic energy stress limits bacterial growth [61]. The consistency in archaeal numbers suggests that due to competition from bacteria, similar resource density may be available to archaea at all depths.

Soil biological diversity is thought to reflect the diversity of microhabitats within the soil structure [62]. In addition to the homogenization of nutrient availability, tillage homogenizes the available microhabitats and can therefore lead to reduced microbial diversity [62,63]. Conversely, increased compartmentalization and limited connectivity in the absence of tillage disturbance can lead to increased species richness [64]. In our study, the diversity measures did not change significantly with depth under standard till treatments, but diversity increased with depth under no-till. This increase in diversity is consistent with restrictions to transport for both nutrients and microbes and protection (sequestration) of C at greater depth [64,65,66].

The decrease in nutrient availability with depth measured directly as decreased soil C, N and OM [11], and reflected by decreases in total bacterial numbers in this study, was also associated with a shift in microbial traits. The decrease in community-weighted mean genome size and 16S rRNA copy number with depth indicated that with increasing depth the microbial communities were enriched for slower growing specialist organisms—the stress tolerators in the C-S-R strategy scheme [31,67,68].

### Effects of tillage

While tillage contributed to distinct distributions of bacterial density at different depths, it did not lead to significant differences in the overall bacterial density in the full 0–30 cm soil column (S2 Fig). This means that the increased C and N concentrations under no-till did not lead to an increase in total microbial populations, likely related to more limited nutrient transport and therefore limited nutrient availability to microbes in the absence of tillage [64]. Archaea also did not show changes in density with tillage, though they are more likely to be limited by competition with bacteria than actual nutrient availability as described above. Conservation tillage has been linked to increases in overall diversity (see e.g. [14,21,63,69,70]) likely related to the preservation of microhabitats [62,63]. Consistent with prior studies, we observed a significant increase in the Shannon diversity index under no till treatment (Fig 2).

While tillage acts to redistribute nutrients throughout the tilled soil column, it does so on a periodic basis, resulting in periodic releases of physically protected organic materials, [13,71] and has been linked to the selection for fast growing copiotrophs [72]. Increases in major soil phyla common in low nutrient environments—such as *Acidobacteria*, *Planctomycetes and Verrucomicrobi*a—have been linked to conservation agriculture, while increases in organisms more common in high nutrient environments, such as *Actinobacteria*, have been linked to conventional tillage [9,73]. We did not observe significant differences in relative abundances of the top phyla based on tillage treatment, but we did observe significant increases in average 16S rRNA copy number under standard tillage. Organisms that quickly mobilize to consume pulses of available nutrients generally possess greater numbers of ribosomal genes [25,26,74]. Tillage therefore appears to enrich for competitors in the C-S-R strategy model [31,33]. By outgrowing other organisms during times of plenty, these competitors may also have reduced the overall diversity of the system or the reduced diversity could have resulted from the reduction in habitat heterogeneity (Fig 2).

### Effects of cover crops

In California’s Central Valley, cover crops are grown during the winter rainy season. Soil samples were collected in late fall, between crop harvest and cover crop planting: i.e. in the period furthest from growing cover crops and therefore when seasonal effects of cover crops were minimized. Consequently, we believe that changes in microbial communities were more likely due to the long term and cumulative effects of cover cropping, such as higher organic C inputs and shorter fallow periods. These conditions create an environment with greater resource diversity and more consistent nutrient supply [75]. The organic C provided by cover crop root exudates and plant residues has been shown to increase biomass and change the composition of soil microbial communities [9,73,76].

Cover cropping led to a significant increase in total bacterial numbers in comparison to NO treatments at all depths (Fig 1), but there was no significant difference in Shannon diversity between cover cropped and NO treatments. Many Archaea associate with plant roots and increases in their numbers have been linked to growing of cover crops in other systems [59,77]. Though archaea also increased in numbers with cover crops in our study (S2 Fig), the magnitude of change in numbers from NO to cover crop treatments was lower for archaea than for bacteria, and may have reflected a limited ability of archaea to compete with bacteria for the same resources [61].

Cover crops also give rise to other changes in the soil environment than just addition of nutrients [75], and altogether these may have favored organisms with a greater metabolic range (i.e. larger genomes) [29]. Also, in keeping with a continuity of resource provision throughout a greater part of the year [9,75,76], cover cropped treatments were not enriched for organisms with especially high numbers of rRNA genes. Rather, moderate copy numbers of ribosome genes and larger genome size (Fig 4) with cover crops indicates that cover cropping appeared to favor moderately-fast growing organisms with greater metabolic range—ruderals in the C-S-R scheme [29,31].

### Utilizing the trait based model

To improve the design of biologically-based farming systems, we need a better understanding of how different management practices interact with soil microbial community development. Assigning microbial taxa into life strategy groups based on their traits (e.g. C-S-R) is an important step toward this goal because these life strategies may ultimately link to differences in function [33].

In addition, our analysis provided valuable information for the potential adaptation of the C-S-R model to agricultural soil microbial communities [31]. While microorganisms at greater depth and under no-till showed a combination of smaller genome size and lower 16S rRNA copy number—as predicted for stress tolerators living under limiting resources—microbial community characteristics did not follow our expectations for competitors and ruderals based on the C-S-R model [30,32]. Specifically, microorganisms under cover crops trended to larger genomes but low 16S rRNA copy numbers—characteristics predicted for ruderals that allow adaptation to a wider range of conditions, while microorganisms under standard till tended to have medium genome size and higher 16S rRNA copy numbers—characteristics predicted for competitors allowing rapid response to resource availability (Fig 5). This suggests that the ruderal-tending communities identified in this study consisted of microorganisms responding to a wider variety of C-inputs rather than to greater disturbance, while competitor-tending communities responded to periodic abundance of resources rather than the associated disturbance. This difference may be due to issues of scale—bacteria and archaea are largely associated with microaggregates [78,79], and thus they may be more protected from physical disruption by tillage than other organisms, such as plants or fungi. In short, whereas ruderal plants find a wider variety of niche growth opportunities associated with disturbance [30], bacteria appear to find a wider variety of metabolic opportunities associated with more diverse primary producers (i.e. plants). Further studies of microbial traits and life strategies may recommend further adaptation of the C-S-R framework to better reflect microbial lifestyles.

## Conclusions

In order to design improved farming systems that provide greater productivity more sustainably, we need to understand how different farming practices affect the soil microbial community. Toward this end, our study provided new insight into the microbial consequences of two widely used conservation agriculture practices. Increased variety and supply of nutrients provided by cover crops likely was responsible for increased microbial abundance, community diversity, and broader metabolic capacities per microbe. In the absence of cover crops, the no-till treatment selected for greater bacterial diversity and slower growing microbes that prefer a more stratified and heterogeneous environment. Soil homogenization under standard tillage in the absence of cover crops led to overall decrease in diversity, likely due to decreases in microenvironments, and selected for fast growing competitors that respond quickly to periodic pulses of nutrients. As we build our understanding of how these different microbial types function, we will improve our ability to sustainably intensify agricultural production using targeted microbial management.

## Supporting information

S1 Fig The effect of different treatments on community-weighted mean estimated traits (rRNA gene copy numbers and genome size) pooled across treatments. Estimates were carried out for a) average 16S rRNA gene copies per genome and b) average genome size. The effects of soil depth, tillage, and cover cropping are shown. Letters above boxplots indicate significant difference (p < 0.05).
(TIF)

S2 Fig Total bacteria and archaea numbers. Total bacteria and archaea numbers in a Mediterranean-climate agricultural soil at different depths and under different cropping regimes: a-b) depth; c-d) tillage; e-f) cover cropping. Letters above boxplots indicate significant difference (p < 0.05); letters in brackets indicate significant difference (p < 0.1).
(TIF)

S1 Table Treatments and plots sampled. (XLSX)

S2 Table Illumina sequencing primers used in this study. (XLSX)

S3 Table Pairwise Spearman correlation coefficients. Coefficients of soil physicochemical data and total bacterial and archaeal numbers in the NTCC, STCC, NTNO and STNO treatments at WSREC. Significant correlations (P<0.05) highlighted in green (positive) or red (negative).
(XLSX)

S4 Table Average values for total bacteria and archaea under different treatments. (XLSX)

S5 Table The relative average abundance of the top 15 bacterial phyla and archeaal phyla under different treatments. (XLSX)

S6 Table The average values for assigned traits rRNA gene copy number and genome size under different treatments. (XLSX)
