# Supplementary figures and images for: Long-term use of cover crops and no-till shift soil microbial community life strategies in agricultural soil
Source: PLoS One. 2018 Feb 15;13(2):e0192953. doi: 10.1371/journal.pone.0192953 (PMC5814021; doi:10.1371/journal.pone.0192953)

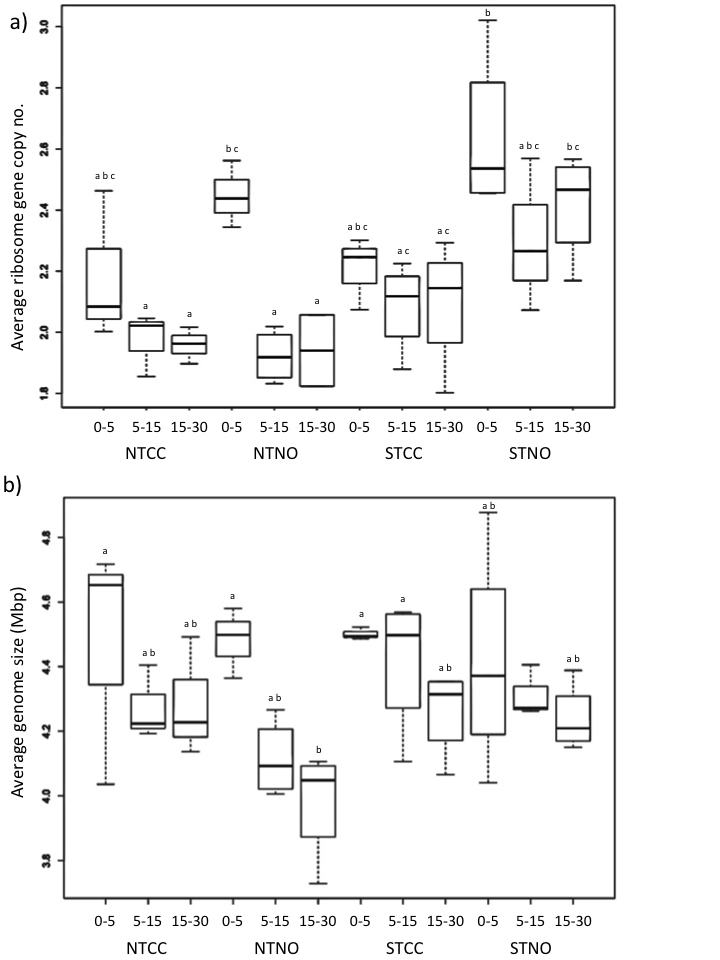

Supplement: S1 Fig — Estimates were carried out for a) average 16S rRNA gene copies per genome and b) average genome size. The effects of soil depth, tillage, and cover cropping are shown. Letters above boxplots indicate significant difference (p < 0.05). (TIF) [file pone.0192953.s001.tif]

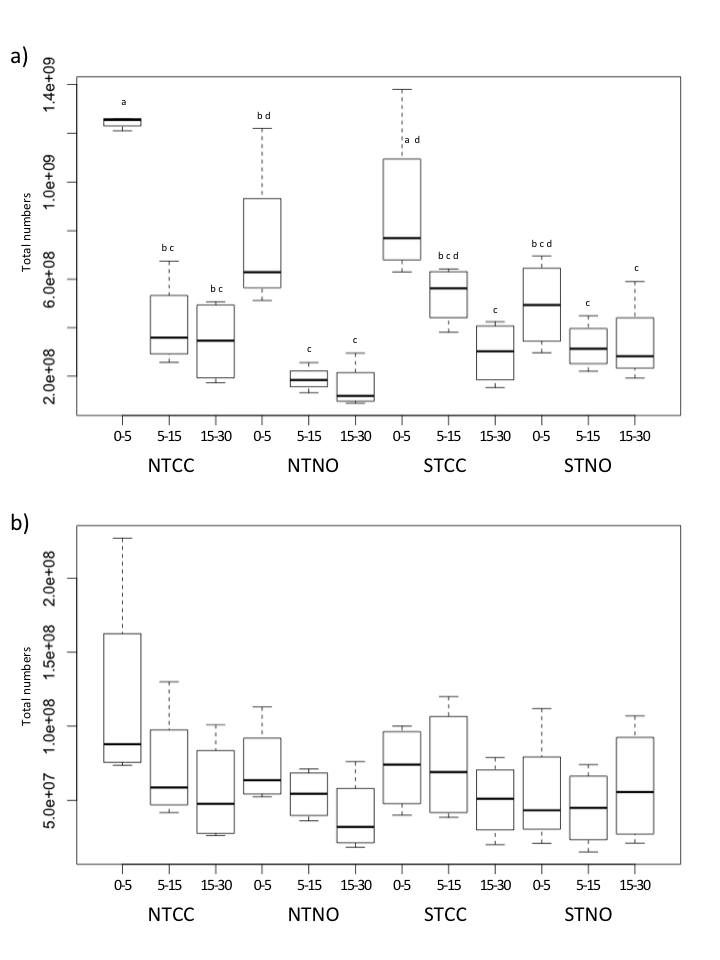

Supplement: S2 Fig — Total bacteria and archaea numbers in a Mediterranean-climate agricultural soil at different depths and under different cropping regimes: a-b) depth; c-d) tillage; e-f) cover cropping. Letters above boxplots indicate significant difference (p < 0.05); letters in brackets indicate significant difference (p < 0.1). (TIF) [file pone.0192953.s002.tif]
